# Agreement between home and ambulatory blood pressure measurement in non-dialysed chronic kidney disease patients in Cameroon

**DOI:** 10.11604/pamj.2018.29.71.12078

**Published:** 2018-01-24

**Authors:** Audrey Manto, Anastase Dzudie, Marie Patrice Halle, Léopold Ndemnge Aminde, Martin Hongieh Abanda, Gloria Ashuntantang, Kathleen Ngu Blackett

**Affiliations:** 1 Clinical Research Education, Networking and Consultancy (CRENC), Douala, Cameroon; 2 Department of Medicine, Douala General Hospital, Douala, Cameroon; 3 Soweto Cardiovascular Research Group and NIH Millennium Fogarty Chronic Disease Leadership Program, Department of Medicine, University of the Witwatersrand, Johannesburg, South Africa; 4 Department of Clinical Sciences, Faculty of Medicine and Pharmaceutical Science, University of Douala, Cameroon; 5 School of Public Health, Faculty of Medicine, The University of Queensland, Brisbane, QLD, Australia; 6 Department of Internal Medicine, Faculty of Medicine and Biomedical Sciences, University of Yaounde 1, Yaounde, Cameroon; 7 Department of Internal Medicine, Yaounde General Hospital, Yaounde, Cameroon; 8 Department of Medicine, Yaounde Teaching Hospital, Yaounde, Cameroon

**Keywords:** Chronic kidney disease, home (ambulatory) blood pressure monitoring, agreement

## Abstract

**Introduction:**

home blood pressure measurement (HBPM) is not entirely capable of replacing ambulatory blood pressure (BP) measurement (ABPM), but is superior to office blood pressure measurement (OBPM). Although availability, cost, energy and lack of training are potential limitations for a wide use of HBPM in Sub-Saharan Africa (SSA), the method may add value for assessing efficacy and compliance in specific populations. We assessed the agreement between HBPM and ABPM in chronic kidney disease (CKD) patients in Douala, Cameroon.

**Methods:**

from March to August 2014, we conducted a cross sectional study in non-dialyzed CKD patients with hypertension. Using the same devices and methods, the mean of nine office and eighteen home (during three consecutive days) blood pressure readings were recorded. Each patient similarly had a 24-hour ABPM. Kappa statistic was used to assess qualitative agreement between measurement techniques.

**Results:**

forty-six patients (mean age: 56.2 ± 11.4 years, 28 men) were included. The prevalence of optimal blood pressure control was 26, 28 and 32% for OBPM, HBPM and ABPM respectively. Compared with ABPM, HBPM was more effective than OBPM, for the detection of non-optimal BP control (Kappa statistic: 0.49 (95% CI: 0.36 - 0.62) vs. 0.22 (95%CI: 0.21 - 0.35); sensitivity: 60 vs 40%; specificity: 87 vs. 81%).

**Conclusion:**

HBPM potentially averts some proportion of BP misclassification in non-dialyzed hypertensive CKD patients in Cameroon.

## Introduction

Blood pressure (BP) control has been widely highlighted as a key component in decreasing the progression of Chronic Kidney disease (CKD) [1] and reducing the overall cardiovascular risk of affected patients [2]. In order to avoid BP control misclassification, a reliable and accurate measurement tool is required, taking into account its performance and the training or expertise of the user [3]. Owing to the relative availability and ease of use, office BP measurement (OBPM) was commonly used in the past for the assessment of BP control in patients with CKD; but over the last decade, several studies have shown that out-of-office BP measurements perform better than OBPM [3, 4], with Ambulatory BP Measurement (ABPM) recognized as the gold standard [3, 5, 6]. In low income settings like most Sub-Saharan Africa (SSA) countries where about half of the population lives on less than a dollar per day [7], the high cost of ABPM compounded by limited accessibility and availability precludes its use [8]. Thus, home BP measurement (HBPM) appears to be an alternative to both OBPM and ABPM. However, the performance of HBPM is influenced by a degree of accuracy which relies on how familiar the patient is with the tool, their mastery of the measuring procedure and the trueness of the values [9, 10]. In SSA, very few patients own BP measurement devices. In rare instances, those who have, either approach nurses to assist with their BP measurements or are likely to wrongly measure their own BP [11, 12]. Moreover, most of the devices commonly used neither have a memory nor a printer system, or are simply invalid [13]. Evidence from developed countries has shown that HBPM is a relatively cheap, reliable and an accurate alternative for the assessment of BP control in patients with CKD [8], however, studies from SSA to support this are scanty. The aim of our study was to determine whether HBPM using an OMRON device provides similar results to a 24-hour ABPM recording in patients with chronic kidney disease in Cameroon.

## Methods

**Study design, setting and sampling**: This was a cross sectional study carried out from March to August 2014 in the nephrology unit of the Douala General Hospital; a tertiary level, referral and teaching hospital located in Douala, the economic capital of Cameroon. Through consecutive sampling, consenting adult patients (age ≥ 18 years) with CKD and hypertension, receiving regular BP lowering medications for at least 14 consecutive days were included. Eligible patients who met the above criteria received explanations in simple terms, about the objectives and nature of the study. Those who freely provided written informed were included. Participants were free to withdraw from the study at any time without their decision influencing their care. We excluded data from patients with inability to obtain HBPM, incomplete HBPM or ABPM and invalid ABPM data. A trained investigator at the outpatient consultation using interviews and patient's record collected data. Sociodemographic and clinical characteristics collected were; sex, age, weight, height, abdominal circumference, smoking, alcohol use, physical activity status presumed aetiology and stage of CKD, duration and treatment of hypertension (number of drugs and their drug-classes), comorbidity such as dyslipidemia and diabetes. The study protocol was approved by the Universite des Montagnes Institutional Ethics Board and received administrative authorization from the Douala General Hospital, Cameroon. The study adhered to the declaration of Helsinki [14].

**Blood pressure measurements**: Each patient had nine office blood pressure measurements, eighteen measures at home (six per day for three consecutive days) and an ABPM over 24 hours.

**Office blood pressure measurement (OBPM)**: After a rest of five minutes and patient in sitting position, three consecutive BP measurements were done on the left upper arm using an OMRON M2 basic sphygmomanometer device. An interval of five minutes was allowed between the different measurements. The mean of the three BP values was considered for the study.

**Home blood pressure measurement (HBPM)**: The procedure was explained to all patients and they were trained with the same sphygmomanometer used for the OBPM. After assessing the ability of each patient to accurately measure their own BP, they were required to record their home BP three times in the morning and likewise in the evening, with an interval of five minutes between the different measurements. This was done during three consecutive days and recorded the values in a diary. For each patient, the home BP value was defined by the mean of the eighteen values.

**Ambulatory blood pressure measurement (ABPM) **: A twenty-four hour ambulatory BP monitoring was performed the day of the OBPM, using the “Eutherapie” device. The daytime and nighttime periods were pre-established; 6:00 am to 10:00 pm and 10:00pm to 6:00 am respectively. The monitor recorded every 15 minutes during the day and every 30 minutes during the night. This was done on a working day and patients were instructed to maintain their routine activities, but stop any movements and keep the arm extended at the time of cuff inflation. The success of the measurement was guaranteed when ≥ 70% of the recordings were valid with one or more valid value per hour.

**Operational definitions**: CKD was defined as the presence of kidney damage of more than three months duration or estimated Glomerular Filtration Rate less than 60 mL/min/1.73m² via the Modified Diet on Renal Disease (MDRD) formula [15], with serum creatinine measured less than 3 months from the BP measurements. The stages of CKD were defined from one to five according to the Kidney Disease Improving Global Outcome (KDIGO) 2012 classification [16]. Office and HBPM targets were 140/90 and 135/85 mmHg respectively. ABPM was considered normal when daytime and nighttime values were respectively less than 135/85 and 120/70 mmHg, with a cut-off of 130/80mmHg for the 24-hour period. White coat effect was defined as lack of controlled office BP or home BP and a normal ambulatory BP. Masked hypertension was considered if the patient had normal office BP or home BP and an elevated ambulatory BP [**6**]. True positive or negative BP were represented by optimal or non-optimal BP control for both office or home and ambulatory BP.

**Statistical analysis**: Data were analyzed using IBM SPSS statistical software v.16 for Windows (SPSS Inc., Chicago, IL, USA). Qualitative variables were summarized as frequencies and percentages. Continuous variables were represented as means and standard deviations (SD). Chi square and analysis of the variance (ANOVA) tests (with equivalents) were used for categorical and continuous group comparisons respectively. Quantitative agreement of ambulatory BP with both office and home BP was assessed using Bland-Altman plots and by calculating bias and limits of agreements (± 2 SD). The qualitative agreement was analyzed using the Kappa statistic, calculation of sensitivity and specificity. Statistical significance was set at p-value less than 0.05.

## Results

**General and clinical characteristics of the study population**: Of fifty patients included, four were excluded from the final analysis because of inability to conduct HBPM (one) and invalid HBPM (one) and ABPM (two). Among the remaining 46 patients included, 28 (61%) were men. The mean age was 56.2 ± 11.4 years. Their clinical characteristics are summarized in Table 1.

**Table 1:** Baseline profile of the study population

Characteristics	TotalN=46, Mean ± SD or n (%)
Age, years	56,2 ± 11,4
Body Mass Index (kg/m2)	27.4 ± 5.0
Obesity	12 (26.1)
Abdominal circumference (cm)	98.0 ± 13.9
Smoker	0 (0)
Sedentarity	27 (58.7)
Diabetes mellitus	28 (60.9)
Dyslipidaemia	
Total Hypercholesterolemia≥ 2.5 g/L	(22.8)
Increase of LDL-c ≥ 1,6 g/L	4 (21.1)
Low HDL-cholesterol (M< 0.4g/L and F < 0.5g/L)	7 (30.0)
Elevated triglycerides ≥ 1.5g /L	5 (26.3)
Low HDL-cholesterol & elevated triglycerides	2 (18.1)
Presumed aetiology of CKD	
Hypertension	16 (34.8)
Unknown	12 (26)
Diabetes mellitus	11 (23.9)
Glomerulonephritis	3 (6.5)
Other	1 (2.2)
Mean Creatinine (mg/dl)	36.3 ± 21.0
Mean estimated Glomerular Filtration Rate (mL/1.73m2)	28.7 ± 23.1
CKD Stages 4 & 5	31 (67.4)
Hypertension Duration (years)	11.7 ± 9.5
BP lowering medications	
Triple BP lowering medication	22 (47.7)
Renin Angiotensin Aldosterone System inhibitors	40 (87)
Calcium channels blockers	40 (87)
Diuretics	30 (65.2)

**Blood pressure control**: The mean office systolic BP (SBP) was 145.2 ± 11.7 mmHg and diastolic BP was 87.9 ± 11.3 mmHg. Isolated SBP control was achieved in 14 (30%) patients and in 27 (58%) patients for the diastolic, while in 12 (26%) patients both were optimal (Table 2). Mean systolic and diastolic home BP were 144.3 ± 17.1 mmHg and 80.9 ± 12.3 mmHg respectively. Mean systolic and diastolic ambulatory BP were 133.1 ± 12.6mmHg and 82.9 ± 8.9 mmHg. Optimal BP was observed in 15 (32%) participants and 19 (41%) for SBP and DBP each other.

**Table 2:** Blood pressure profile of the study population

Office Blood Pressure Measurement (OBPM)			Home Blood Pressure Measurement(HBPM)			Ambulatory Blood Pressure Measurement (ABPM)		
BP variables (mm Hg)	Optimal BPMean±SD orn (%)	Non-optimal BPMean±SD orn (%)	BP variables(mm Hg)	Optimal BPMean±SD orn (%)	Non-optimal BPMean±SD orn (%)	BP variables (mm Hg)	Optimal BPMean±SD orn (%)	Non-optimal BPMean±SD orn (%)
Systolic	131.9 ± 6.7	158.5 ± 16,7	Systolic	124.0 ± 8.5	164.6 ± 25.7	Systolic	120.5 ± 5.1	146.2 ± 20.1
Diastolic	80.1 ± 6.7	95.7 ± 15.9	Diastolic	72.7 ± 7.6	89.1 ± 17.0	Diastolic	74.2 ± 4.5	91.6 ± 17.8
Systolo-diastolic (140/90)	**12 (26.1)**	34 (73.9)	Systolo-diastolic (<135/85)	**13 (28.3)**	33 (71.7)	Systolo-diastolic (<130/80)	**15 (32.6)**	31 (67.4)
Systolic (<140)	14 (30.4)	32 (69.6)	Systolic (<135)	14 (30.4)	32 (69.6)	Systolic (<130)	19 (41.3)	27 (58.7)
Diastolic (<90)	27 (58.7)	19 (41.3)	Diastolic (<85)	27 (58.7)	19 (41.3)	Diastolic (<80)	19 (41.3)	27 (58.7)

BP: blood pressure; expressed in millimeters of mercury

**Figure 1:**
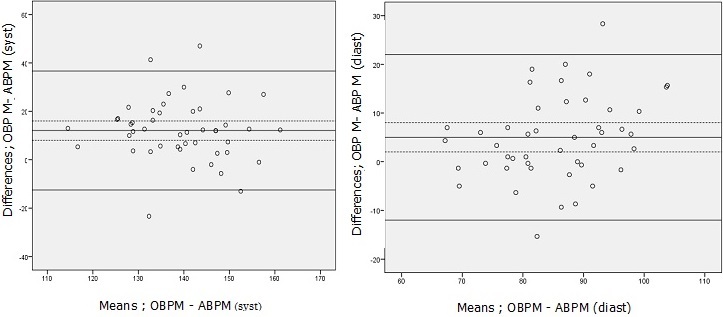
Bland-Altman plots showing agreement between OBPM and ABPM both systolic and diastolic

**Figure 2:**
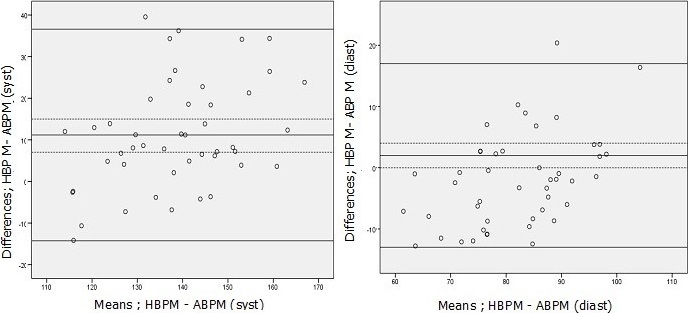
Bland-Altman plots showing agreement between HBPM and ABPM both systolic and diastolic

**Quantitative agreement**: Considering ABPM as the standard, the mean overestimation by clinical measurement was 12% for SBP and 5% for DBP. The HBPM averagely overestimated the BP by 11% for the systolic and 2% for the diastolic. With respect to the mean office BP, the Standard Deviation of the Differences (SDDs) was respectively 17.4 mmHg and 4.4 mmHg for systolic and diastolic BPs. According to the means of HBP, the SDDs were 15.8 mmHg and 1.6 mmHg for systolic and diastolic respectively (Figure 1, Figure 2).

**Qualitative agreement**: The office BP measurement Kappa index was poor (K = 0. 22 (95% CI: 0.21-0.35)) whereas the HBPM was moderate (K = 0.49 (95% CI: 0.36-0.62)). Overall, the percentage of patients with optimal BP control was six (13%) with both clinic and ABPM and nine (19%) with both home and ABPM. Higher rates (25% for office and 58% for home) were observed for uncontrolled BP values. The prevalence of masked hypertension was 13% (n = 6) and 4% (n = 8) for office and home respectively, while white-coat effect was 19% (n = 9) and 13% (n = 6) for office and home measured BPs (Table 3). Sensitivity and specificity was 40 vs. 60 % and 81 vs. 87% for office and home BP respectively (Table 4).

**Table 3:** Qualitative agreement: Kappa index

**Office BP**	**Ambulatory BP**			**Kappa K**	**p-value**
	**Optimal**	**Non optimal**	**Total**	**0.22**	013
Optimal	6 (13.0)	6 (13.1)	12 (26.1)		
Non optimal	9 (19.6)	25 (54.3)	34 (73.9)		
Total	15 (32.6)	31 (67.4)	46 (100)		
**Home BP**	**Ambulatory BP**				
	**Optimal**	**Non optimal**	**Total**	**0.49**	0.001
Optimal	**9 (19.6)**	4 (8.7)	13 (28.3)		
Non optimal	6 (13.0)	**27 (58.7)**	33 (71.7)		
Total	15 (32.6)	31 (67.3)	46 (100)		

**Table 4:** Qualitative agreement: sensitivity and specificity

	Office BP	Home BP
TRUE POSITIVE	6	9
FALSE POSITIVES	6	4
TRUE NEGATIVES	25	27
FALSE NEGATIVES	9	6
SENSITIVITY	0.40	**0.60**
SPECIFICITY	0.81	**0.87**

## Discussion

In this study assessing the degree of agreement between HBPM and 24 hour ABPM in patients with CKD in Cameroon, we found only a quarter of patients had controlled BP using office measurement. Optimal BP control increased to about a third of participants using HBPM or ABPM. We also established that home BP had better agreement with ABPM compared with office BP with higher sensitivity and specificity. Our findings correlate with previous studies revealing the low prevalence of BP control in patients with CKD [17-19]. Optimal BP control increased from office (26%) through home (28%) to ambulatory (32%) BP measure, further confirming the superiority of ABPM in the management of hypertension among these patients [20]. By using ABPM as the gold standard, close values were observed when analyzing quantitative agreement through bias (12% for systolic and 5% for diastolic for OBPM vs. 11% for systolic and 2% for diastolic for HBPM) and SDDs (17.4/4.4 mmHg for OBPM vs 15.8/10.6 mmHg for HBPM). We used standardized OBPM because this measure can be easily improved by healthcare professionals [21]. In fact, over four-fifths of our patients had never conducted HBPM. Although some patients found it relatively challenging to assimilate all details of the BP measurement procedure during training, the “stress” due to the white coat and hospital environment was reduced but replaced by other sources (fear of failure of the measurement procedure due to the lack of acquaintance, fear of error in recording) which are potential source of misreporting [22, 23]. Agarwal et al found more elevated differences (bias: 13 and 12.5% for OBPM vs 7.5 and 4% for HBPM and SDDs: 19.3/10.3 mmHg for OBPM vs 13.6/7.2 mmHg for HBPM) [20]. The wide utilization of HBPM in developed countries and measurement duration of seven days (as opposed to three days in our study) could have played an important role in this difference. However, due to differences in BP target values from OBPM to HBPM (140/90 vs 135/85 mmHg), there is need for more emphasis on qualitative agreement, that is, the ability of either method to categorize a patient as controlled or not, in accordance with the ABPM classification. When comparing OBPM and ABPM, we obtained similar classification in 67% of our patients, representing a poor Kappa index of 0.22. This poor performance of OBPM was previously reported by Gorostodi et al. in Spain [24]. The HBPM Kappa index increased to moderate level with K= 0.49 but remained less than 0.5.

The agreement in controlled patients was lower (13% for OBPM and 19% for HBPM) compared to that of uncontrolled ones (54 % for OBPM and 58% for HBPM). This is in line with the observed high specificities (81% for OBPM and 87% for HBPM) than sensitivities (40% for OBPM and 60% for HBPM), suggesting that HBPM is more accurate when classifying a patient as uncontrolled and highlighting the use of ABPM when BP seems optimal. This is similar to the findings in children with CKD [25] and in contrast to findings presented by Agarwal et al in which sensitivity and specificity for HBPM were both > 80% [20]. As previously mentioned, the training of the patients and the difference in duration of the HBPM (three vs seven days) could partly explain these differences. Similar variations are observed from a systematic review of 20 studies in the general population (specificity: 56.7% and sensitivity: 83% for HBPM) [26]. The heterogeneity as well as differences in study design and target populations of studies included in this review likely explains this difference. Discordances (high BP in OBPM/HBPM and normal BP in ABPM, or the contrary) are frequent causes of over or under treating patients and HBPM may contribute to avoid these errors [6]. Our study suggests that HBPM potentially reduces white-coat hypertension in 7% of the cases (20% with OBPM vs 13% with HBPM), which is similar to findings obtained from developed countries (30% with OBPM vs 24% for HBPM) [20, 27]. In a like manner, prevalent masked hypertension is equally reduced to 5%, which is slightly less than estimates from developed countries [28, 29]. This demonstrates the ability of HBPM to avert a significant proportion of likely misclassified patients from office BP measurement. Our study had some limitations. The duration of measurement of home BP was three days, which is shorter than seven days suggested by other authors [8]. However, our strict adherence to standard procedure guidelines as well as the training provided to study participants would possibly provide confidence in the accuracy of our measurements. Secondly, our study sample was relatively small with a consequent impact on study power. However, we attempted to include all possible eligible participants in the study center. Considering that it was a single center study in Cameroon, our findings (in terms of generalizability) should be interpreted with caution. However, it should be noted that in Cameroon like most other countries in SSA, ABPM is expensive with limited availability, while HBPM is not widely used. Our study explored the degree of accuracy of a comparatively affordable measure (HBPM) in patients with CKD, for whom thorough BP control is a key pillar in management and prognosis. Further to this, to enhance accuracy of our results, patients were trained on how to measure their BPs accurately with standard devices and guidelines.

## Conclusion

Our study suggests that HBPM is potentially a reliable alternative to ABPM for the assessment of BP control in Cameroonian patients with CKD. Its accuracy over clinic or office BP measurement is in line with reports from western countries. While we advocate for a possible wider application of HBPM in SSA settings, larger multicenter studies are warranted to confirm these findings.

### What is known about this topic

- ABPM is the gold standard for assessment of BP control in non-dialyzed patients with hypertension;
- Its utilization is limited in SSA due to limited availability and cost;
- HBPM is a reliable and cheap alternative but in SSA, lack of training of patients and wide use of invalid devices are potential limitations.

### What this study adds

- HBPM is a reliable alternative in Cameroon;
- Its accuracy is higher in case of non-optimal BP;
- Wider use of HBPM in SSA could potentially increase its accuracy.

## Competing interests

Authors declare no competing interests.
